# The science of food security

**DOI:** 10.1038/s41538-018-0021-9

**Published:** 2018-08-06

**Authors:** Martin Barry Cole, Mary Ann Augustin, Michael John Robertson, John Michael Manners

**Affiliations:** CSIRO Agriculture and Food, Australia, 11, Julius Avenue, North Ryde, New South Wales 2113 Australia

**Keywords:** Business and industry, Environmental sciences, Biotechnology, Agriculture, Developing world

## Abstract

We need to feed an estimated population in excess of 9 billion by 2050 with diminishing natural resources, whilst ensuring the health of people and the planet. Herein we connect the future global food demand to the role of agricultural and food science in producing and stabilising foods to meet the global food demand. We highlight the challenges to food and agriculture systems in the face of climate change and global megatrends that are shaping the future world. We discuss the opportunities to reduce food loss and waste, and recover produce that is currently wasted to make this the new raw ingredient supply for the food industry. Our systems-based perspective links food security to agricultural productivity, food safety, health and nutrition, processing and supply chain efficiency in the face of global and industry megatrends. We call for a collaborative, transdisciplinary approach to the science of food security, with a focus on enabling technologies within a context of social, market and global trends to achieve food and nutritional security.

## Framing the food security challenge against global megatrends

**Fig. 1:**
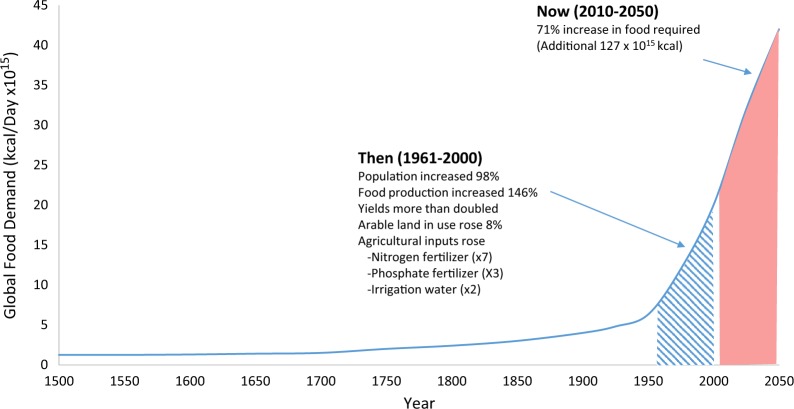
Framing the food security challenge (adapted from Keating et al. 2014; Keating and Carberry, 2010)^2,3^

Feeding the world sustainably is one of our society’s grand challenges.^1^ An exponential rise in population between 1961–2000 increased the demand for food. The demand was met by a combination of scientific and technological advances, government policy, institutional intervention and business investment, innovation and delivery. However increased farm inputs and outputs were partly at the expense of detrimental effects on the environment.^2,3^ In 2050, it is estimated there will be 9.7 billion people, and we will require about 70% more food available for human consumption than is consumed today (Fig. 1).

**Fig. 2:**
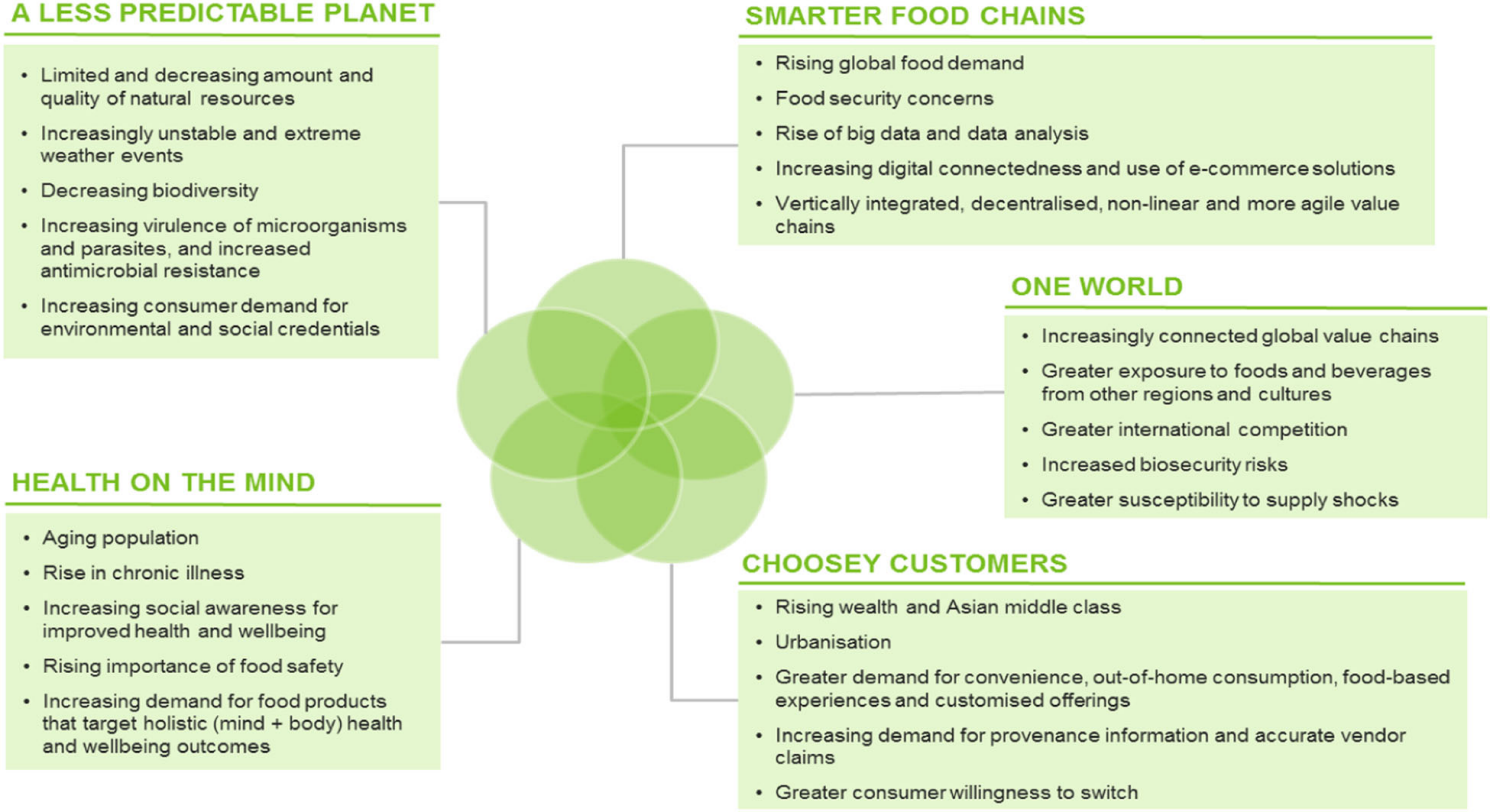
Key drivers and potential impacts arising from global megatrends in Food and Agriculture (Adapted from Hajkowicz and Eady, 2015; CSIRO Futures, 2017)^5,6^

A megatrend is defined as a substantial shift in social, economic, environmental, technological or geopolitical conditions that may reshape the way a sector operates in the long-run.^4^ Hajkowicz and Eady (2015)^5^ identified five megatrends evident in global food and agribusiness that will have a significant impact on the sector over the next 20 years. The key megatrends are summarised in Table 1. The potential impacts of the megatrends within the food and agribusiness sector are highlighted in Fig. 2.^6^ These and other trends including diminishing natural resources, urbanisation, growth of megacities, changing demographics and shifting dietary patterns will have a significant effect on security. FAO has recently called for a transformative change to agriculture and food systems.^7^

**Table 1:** Megatrends in food and agribusiness^5,6^

Megatrend	Consequences
A less predictable planet	Supply of limited resources is being further constrained by more severe and unpredictable climate events and more potent microbes, pests and diseases, causing food producers to more seriously consider the environmental life cycle impact of food production activities.
Health on the mind	An ageing population, rising levels of chronic disease and increasing social awareness around health and wellbeing are creating demand for foods that provide specific and holistic health outcomes.
Choosey customers	Rising wealth, increasing choice and greater market access are driving demand for a more diverse range of foods and food service options that are tailored to individual preferences and lifestyles.
One world	As food and beverage value chains become increasingly global, new market opportunities are created while at the same time introducing competition and supply resilience risks in a volatile world.
Smarter Food Chains	Increasing demand for food, the use of big data and more sophisticated e-commerce platforms are driving the creation of leaner, faster, more agile and low waste value chains.

## Framing the food security solution

**Fig. 3:**
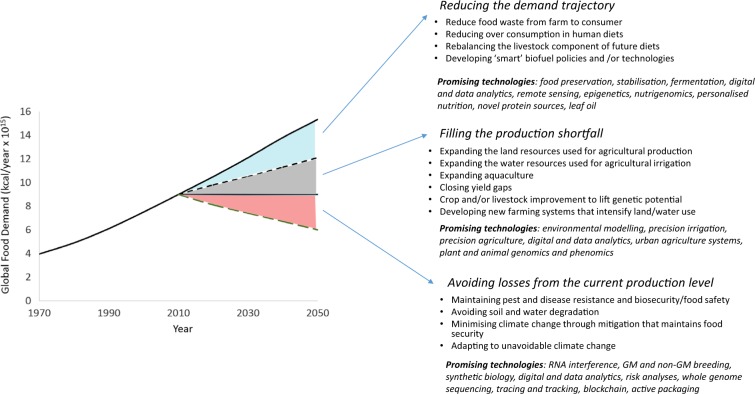
Food wedges framework linking food demand to likely stabilisations and promising technologies (Adapted from Keating et al. 2014)^2^

A previous framing of the food security solution suggested that taking advantage of the advances in agriculture and reducing waste whilst addressing shifting diets, enabled a doubling in agricultural production and a reduction in environmental impacts.^8^ Keating et al. (2014)^2^ developed a simple framework of wedges and modelled the kcal requirement for the growing world population. They suggested the likely approaches or stabilisations that might be needed to deliver food security in terms of reducing demand, filling the production gap and avoiding losses from the current production level (Fig. 3). In this perspective, we use the wedges concept to consider the role of science and the most promising technological approaches that will be required to deliver food security in a resource-constrained environment. We also offer a perspective on the likely impact that global megatrends will have on these endeavours, and consider the need for new ways of working to respond to these trends.

### Unlocking pathways to reduce the food production demand

#### Reducing food waste from farm to consumer

Reducing food wastage, which comprises food loss and food waste, and capturing more of the food that is produced for human consumption is an obvious opportunity to increase food security without increasing the environmental burden of production. Food loss is the decrease in edible food mass, which occurs at production, postharvest and processing stages in the food supply chain, while food waste refers to what is lost at retail and by consumers.^9^ Recovering food loss and waste is a huge opportunity to reduce production demand, given that about 1.6 billion tonnes of food is wasted along the chain and of this 1.3 billion is edible.^9^ The relative amounts of food loss and food waste in various regions vary. Food loss is the major contributor to food wastage in developing countries. This is in contrast to developed countries where waste primarily occurs at the retail and consumer end of the food supply chain.^10^

Food science and technology has a significant role to play in achieving food and nutrition security.^11^ Food preservation and stabilisation technologies to extend shelf life of products (e.g. processing techniques such as drying to reduce water activity, heat treatment or high pressure processing to reduce microbial load or fermentation to reduce pH) underpin the ability of food to be made accessible and safe and are integral to the sustainability of the food supply and reducing food waste.^12^ Good post-harvest handling practices from farm to retail, including supporting logistics and infrastructure, can mitigate against the loss of fresh produce. This is becoming increasingly relevant as the food produced in rural areas has to reach the growing population in urban areas and megacities. This results in increased pressure for the optimisation of the distribution of food flows, improved access to appropriate modes of transportation, infrastructure, and better management of cool chain logistics, to ensure sustainable food supply.

In terms of processing, new extraction technologies such as ultrasound can improve the recovery of oil from biomass.^13^ Natural preservation through fermentation^14^ and separation technologies, such as forward osmosis‚ offer the potential to create new value-added food ingredients and bioactives from food loss and food waste. The preferred option for improving food security is to recover and rescue food loss and food waste for human consumption.

Food banks have been set up in various countries to rescue and redistribute nutritious foods to vulnerable groups. These initiatives reduce food waste, whilst alleviating food insecurity. However, there may be competing interests with various players along the chain who wish to address economic, environmental and social impacts of food wastage. A holistic approach taking into consideration multi-stakeholder perspectives is required to ensure sustainable production and consumption and a win-win solution for all.^15^

Consumers are likely to continue to demand more transparency about the environmental credentials and provenance of food. Digital technology is increasing the access to information about food. The internet of things will be an enabler for digital disruption, leading to leaner production and supply chains. Integration of digital platforms with real-time analytics and sensors for informed decision making could be combined to develop a future node in the food value chain (FOOD LOSS BANK^TM^) to reduce food loss.^16^

There are significant amounts of food loss and waste, by-products and side streams of processing (e.g. straw, leaves and stems, effluents from processing) that are currently diverted to other uses such as animal feed and for the production of chemicals, composting and energy, or being dumped as landfill. It is beyond the scope of this paper to consider these alternative uses of food loss and food waste for non-food purposes.

#### Reducing over consumption in human diets

The food wedge framework considered the future food demand in terms of calories in order to simplify and communicate the likely stabilisations that would be required. In practice though, we also need to consider food demand in terms of providing the diet that will support our future nutritional and health requirements.^17,18^ New metrics based on 'nutritional yield' have been proposed to replace 'tonne/hectare yield' to take into account the importance of demand for nutritious food for sustainable agricultural intensification.^19^ Ironically, small farms that offer more nutritional diversity^20^ may not be in position to afford the new technologies, such as hybrid seeds and genetically modified organisms (GMOs), needed to support intensification.

**Fig. 4:**
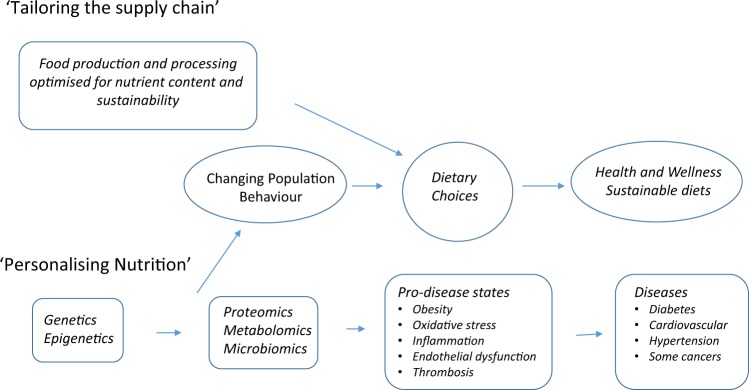
Research strategies for improving public health through better dietary choices and a systems nutrition approach (Adapted from Lewis and Burton-Freeman, 2010 and Kaput et al. 2015)^21,22^

Nutritional food security is complicated by the fact that we need to increase the amount of available food; but at the same time there are over 2 billion people who are obese or overweight. Reducing over consumption in this population represents a significant opportunity to increase food security without having a negative impact on the environment, and at the same time reducing the impacts of the global health burden due to poor diets. There are recommended dietary guidelines available, but these may not be adhered to. A change in consumer behaviour through education combined with the increased availability of healthier processed foods that meet personal needs is required.^21^ The opportunity will be to use a systems approach to nutrition^22^ to tailor the food supply chain to enhance the nutritional content of food matched to personalised nutritional needs whilst also taking into account environmental impacts (Fig. 4). There are exciting new developments in our understanding of the molecular basis of nutrition and obesity, which will lead to new biomarkers for health and wellness. For example, we have a new appreciation for the role of epigenetics in obesity.^23^ Advances in data analytics offer the potential to link new biomarkers based on epigenetics, nutrigenomics, nutritional proteomics, and nutritional metabolomics to agricultural genomics in a more integrated approach to personised nutrition.^24^

However, whilst healthy foods and information may be made available to consumers to make informed choices about food, they may not always make healthy food choices. What consumers eat is governed by a complex interplay of other factors including appetitive behaviours controlled by neural circuits and hormones,^25^ cognitive factors, sensory properties and the feelings of satiety and satiation that the food offers.^26^ An integrated transdisciplinary approach is required to design culturally acceptable foods and optimise healthy food choices for various ethnic populations and religious groups (e.g. Halal and Kosher foods).

#### Rebalancing the livestock component of future diet

Sustainable diets need to protect biodiversity and the environment, optimise natural resources, be culturally acceptable, accessible and affordable to various populations, whilst being safe and nutritious.^27^ The changing dietary patterns and the rise of the middle class have increased the demand for animal products. However, the carrying capacity of land for different diets varies, with it being generally higher for diets with less meat.^17^ The shifting consumption patterns towards lower meat consumption, observed in some developed countries, is a strategy to reduce loss of biodiversity and to offset the effects of climate change.^28^ However, while there is a sustainability driver to move away from meat protein, most consumers are not willing to reduce meat consumption because of ecological reasons alone.^29^ In-vitro cultured meat may be technically feasible to produce but production is currently cost-prohibitive. In addition, the technology faces challenges in overcoming consumers’ willingness to try.

With the increasing population, new sustainable sources of protein from non-animal sources need to be developed. Cereals have, and are expected to continue to be a major source of plant-based protein. Pulses are an emerging source and becoming more prominent with improvements in production practices. Pulses are attractive alternatives from a health perspective as they are rich in proteins, fibre and micronutrients. Algal biomass offers promise as a renewable source of protein, but the economics of production currently limits the growth of the industry.^30^ Improved sea-borne production systems may enable the future growth of plant-based foods.

Insects are sustainable sources for food and feed protein. Improved production systems for edible insects are required to ensure long term sustainability.^31^ Insects have been consumed by some populations (e.g. in South America and Asia), but there has been resistance by Western populations who have a cultural aversion to consumption of insect-based food. Education about social impact may help motivate some to try, but this needs to be accompanied by new product development to improve consumer appeal and sensory quality for different cultures.^32^

#### Developing ‘smart’ biofuel policies and /or technologies

Moving away from first generation biofuels that use highly arable land (i.e., feedstocks such as corn, sugarcane) to second generation biofuels from marginal land or waste (i.e. cellulosic material) may alleviate some of the tension between food or fuel use.^33^ The issues between land, food and energy and the multiple end-use of crops make it greater than just the food versus fuel debate, as their interdependencies should be taken into account when framing land use change policies.^34^ New technologies may offer the potential to produce biofuels from the non-edible parts of plants. Plants do not usually produce oils to any significant levels in their leaf tissues. New technology^35^ allows plants to produce significant levels of oil in their leaves, which may offer a new high yielding source of sustainable biofuel. Levels of >30% oil in tobacco leaf has been achieved using metabolic engineering.^36^

### Unlocking pathways to increase food production

#### Expanding the land resources used for agricultural production

Given that options for unlocking new arable land are limited it is critical that when opening up new land there must be the accompanying infrastructure (e.g. for capturing and storing the rainfall) to avoid its loss through transpiration from the soil. It is also necessary to take into account the significant drop in the water table over the years which results in degradation of the productive environment. Both the removal of forest due to urbanisation and climate change affect land surface evapotranspiration, with climate change having the greater effect than change in land cover usage.^37^

#### Expanding the water resources used for agricultural irrigation

Water security is becoming a global issue. Better forecasting of soil moisture and requirement of crops for water and efficient use of irrigation water may be achieved by combining weather predictions and hydrological modelling, supported by data using new technologies for environmental monitoring and Earth observations from space.^38^ Real-time irrigation smartphone apps and soil-water sensors are also becoming more available to provide advice for optimal irrigation scheduling.^39^ These developments are a step towards precision irrigation to conserve water and maximise water use efficiency.

#### Expanding aquaculture

Aquaculture is the fastest-growing animal food producing sector in the world. The global growth of aquaculture is expected to continue to meet the estimated demand for an additional 40 million tonnes of aquatic food by 2030 to maintain the current per capita consumption.^40^ Sustainable production practices including moving away from fish-based feeds towards those based on plant products, and environmentally-sensitive development that minimises impacts on coastal ecosystems are required. Intensive aquaculture needs technologies that reduce the risk of mass mortalities due to disease. These include rapid, high throughput disease screening of hatchlings, enhanced selection for disease tolerance, production and distribution of better diets using more sustainable ingredients and improved environmental management of production ponds and adjacent environments.^41^

Whilst aquaculture in coastal areas are cost-effective operations, there are undesirable consequences for the environment, biodiversity (e.g. species loss, risk of mangrove extinction) and coastal communities.^42^ Indoor aquaculture with intensive recirculating aquaculture systems mitigate some of risks associated with outdoor aquaculture. Whilst more expensive, there are commercial land-based operations, such as for production of salmon and Rainbow trout. In addition, advances in technology and the use of aquaponic systems enable the culture of exotic species for target customers.^43^

#### Closing yield gaps in existing crop and livestock production systems

Substantial 'yield gaps', the gap between farm and attainable yields, exist in all production systems.^44^ Production advances will be about improving the adoption of existing technology as well as promoting the development of new technology. Advances in digital technologies are enabling precision agriculture that will integrate controlled release fertilisers, pest and weed management, new crop and animal genotypes, soil amelioration techniques and weather and climate forecasting. Modelling to obtain more reliable estimates of magnitude, spatial and temporal variability of yields will help to identify the exploitable yield gap and is a step towards reducing yield gaps.^45^

#### Crop and/or livestock improvement to lift genetic potential

Advances in production per hectare will be underpinned by new genetics tailored to management technologies. The increase in genetic potential will be achieved by selecting genotypes for traits with greater resource use efficiency and tolerance to biotic and abiotic stress through access to novel genetic diversity, deployment of targeted biotechnology and tools to improve confidence in phenotyping and environmental characterisation. Novel technology packages such as more timely sowing systems in crops will be enabled by improvements in pest management, seasonal climate forecasting, information and communication, technologies, and weather monitoring and soil sensing.^46,47^ As yield gaps are closed by better management there is a great imperative to lift genetically programmed yield potential for further gains. In crops, there is a focus on lifting radiation use efficiency to break photosynthetic ceilings. One ambitious approach is to build the highly efficient C4 photosynthetic pathway that operates in crops, such as maize and sugarcane into less efficient C3 plants, such as rice and wheat.

#### Developing new farming systems that intensify land use/water use

Improved agricultural water practices will lead to gains in global crop production. This may be achieved by expanding irrigation by reducing non-productive water consumption, through improved crop water management.^48^ This includes use of techniques to reduce soil evaporation, capture surface run-off, and to improve soil infiltration capacity and efficiency of irrigation systems.

Urban agricultural systems such as urban orchards, roof-top gardens and vertical farming on facades of building and peri-urban agriculture contribute to intensified land use and raises awareness of food production systems in cities. Well-managed urban agriculture reduce greenhouse gases and urban heat.^49^

### Unlocking pathways to avoid losses or future production potential

#### Maintaining pest and disease resistance and biosecurity/food safety

Weeds, pests and diseases cause major losses to current agricultural production systems. Pests and pathogens of crops and livestock are continually evolving and ongoing protection programs are necessary to both maintain current productivity as well as securing further gains. There is pressure to reduce the use of chemical herbicides, pesticides and antimicrobials in agriculture and alternative technologies are needed. The use of genetic approaches such as selective breeding, hybrid seeds and the addition of exogenous genes via genetic modification has been extremely important in increasing yields and reducing chemical inputs in a number of farming systems (e.g. Bt cotton and maize crops). Similarly, novel disease resistance strategies include the cloning and introduction of durable genes^50,51^ and their transfer into other crops to obtain broad resistance, and gene editing to alter susceptibility genes.^52^ There are likely to be many opportunities and challenges that cannot be addressed without GMO technology as climate change harshens farming conditions and global biosecurity threats evolve. However, largely because of consumer acceptance issues, and the high costs of deregulation, the commercial use of GMO technology in a number of countries is limited. Due to these trends there is renewed commercial interest in new non-GMO breeding techniques, such as gene editing, which provides more precision than GMO technology. Another alternative technology is the exogenous application of RNA interference (RNAi) molecules to specifically silence genes in plants and animals. Exogenous RNAi may be used to control viral^53^ and fungal diseases in plants.^54^ Co-application of RNAi with a herbicide can target weed resistance mechanisms and make pesticides more durable. Maintaining pest and disease resistance in crop varieties will need enabling technologies and ecosystem actions to be applied in an integrated manner.

The global food supply chain is extremely complex and many biosecurity issues are also food safety issues. With a large proportion of emerging human infectious diseases originating from animal sources there is an increasing need to consider both animal and human health as a ‘one health’ issue.^55^ Biosecurity and food safety issues may cause a disruption to the food supply chain through direct public health impacts, through recalls or even market ‘avoidance’ of particular trading areas due to real or perceived public health concerns.

In an environment of global interdependence in food safety, countries cannot solely rely upon their own food safety managements systems and it is therefore essential that food safety standards are universally based on sound scientific principles and focus regulatory efforts on genuine public health risks. The global increase in the number of incidents related to food safety in recent years has led to a paradigm shift in the way that food safety is managed. Regulatory efforts have become focused on the use of risk assessment tools to drive food safety policy and standards away from prescriptive to outcome-based control measures. New risk management approaches have been developed that are based on concepts such as of Food Safety Objectives and Performance Objectives.^56^ These approaches enable the food industry to meet specific objectives through the application of the principles of Good Hygienic Practice (GHP) and Hazard Analysis Critical Control Point (HACCP). This modern approach to assuring the safety of the food supply provides a scientific basis that allows industry to select and implement control measures specific to its operations, and also leads to a better understanding of the role of microbiological criteria in testing.^57^

Despite the availability of food safety protocols and the stated intent of companies to implement food safety measures, there are incidences of food recalls and foodborne outbreaks. These may be linked to the lack a good food safety culture. Improving food safety culture requires a high level of senior management commitment to food safety and a shared purpose in maintaining food safety standards amongst employees.^58^ The role of government and food safety audits for compliance are ingredients for reducing risks for foodborne illness.^59^ Promoting good food safety culture through the supply chain should also be supported by Government initiatives at national and international levels.

Global trends including climate change, a growing and aging population, and urbanisation place new demands on producers, manufacturers, marketers, retailers and regulators to ensure food safety. The internationalisation of the food chain has improved food accessibility but this increases the risk of foodborne disease burden.^60^ The spread of microbial resistance due to the use of antibiotics in production and antimicrobials for feed and food preservation is a concern in the food industry. Foods can be carriers of antibiotic-resistant bacteria and enter into the food chain.^61^ Climate change will also present particular challenges to maintaining food safety. There are increased risks due to changes in temperature and in contaminants’ transport pathways.^62^ Advances in science and technology such as whole genome sequencing, active food packaging (e.g. with embedded natural anti-oxidants or anti-microbials), developments in tracing and tracking technologies, information computing technology and big data analysis have the potential to help mitigate the challenges and meet demands.^63^

#### Avoiding soil and water degradation

Technologies and farm practices that maintain groundcover to minimise erosion and nutrient runoff will be important. Precision agriculture techniques will enable reduced use of inputs of agri-chemicals and water that match supply with demand and limit losses. Technologies that can predict and adapt to volatility such as climate fluctuations will be needed.

Soil degradation decreases nutrient potential of soils. Even with tillage practices there is a decrease in fertility. An emerging approach to reducing fertiliser requirements is by reconstituting the nitrogen fixing function in plant cells. This approach relies on using synthetic biology for direct engineering of nitrogenase into the mitochondrial matrix of plants.^64^ While this technology needs to be further developed, it is one that holds promise for the future.

#### Minimising climate change through mitigation that maintains food security

There is currently no global target for greenhouse gas emission mitigation from agriculture. A recent analysis,^65^ for the first time, calculated that in order to limit global warning in 2100 to 2 °C above pre-industrial levels, annual emissions from the agricultural sector must be reduced by 1 gigatonne of carbon dioxide equivalents per year by 2030. Currently available interventions, such as sustainable intensification of dairy production and alternate wetting and drying in irrigated rice, to achieve emission efficiencies will be necessary. Yet these are insufficient, to achieve these targets. There is a need to develop and implement transformative technical options, such as methane inhibitors in the livestock sector, nitrogen inhibitors in annual crops, and innovative policies to promote sequestering soil carbon.

#### Adapting to unavoidable climate change

Given that climate change is now unavoidable and farmers are already living with its impacts, adaptation to the on-going effects will be inevitable.^66^ A number of studies have highlighted that simple changes to management and adoption of existing technology (e.g. change of sowing date, crop mix on farm, irrigation) can negate the modest short-term to medium-term negative impacts of climate change.^67^ However, beyond this time frame more transformative changes to farming systems will be required such as changes to business structure, portfolio management, off-farm investments and geographical diversification.^68^ Breakthrough innovation for increasing photosynthetic potential,^69^ radiation use efficiency or modifying canopy architecture^70^ will be some approaches that may be applied to increase yield potential.

## Perspectives on social challenges

Even when science provides the technical solutions, the adoption of the technologies by stakeholders is not possible without attention to social, consumer and market challenges. The social relevance of research strategies should embed new approaches for evaluating research and innovation, which connect with broader societal values.^71^ The public dialogue around global food security should include consideration of the ethical trade-offs between societal decisions around choice of food for adequate nutrition and environmental sustainability.^72^ Influencing consumer behaviour to choose foods with low environmental footprints, eat appropriate foods in line with nutritional requirement and not to waste food consumption has potential to improve global food security and the sustainability of the food supply.^73^

## Transdisciplinary practice to address food and nutrition security

Agricultural, food, nutritional, economics and social sciences all need to come to bear on the solution for food and nutrition security, because of their multi-faceted interdependencies in the global system and their collective impact on nutritional security.^74^ It is also essential to explore how innovation from other disciplines (e.g. data science, robotics, artificial intelligence, nanotechnology) may impact on food security. Success in achieving food and nutrition security requires an integrated transdisciplinary approach across diverse lines of enquiry.^75^ Addressing food and nutrition security requires consideration of the food ecosystem in its entirety. Ecological and social-institutional approaches are needed because agricultural systems are complex adaptive systems across multiple scales.^76^ This whole of systems initiative for food and nutrition security has to involve a collaborative and transdisciplinary approach^77^against an evolving future of social, market and global megatrends.

## Conclusions and recommendations

It is clear that the food security challenge is complex‚ requiring a focus on both human and planetary health. An integrated system of interventions underpinned by transdisciplinary research and technological innovation will be required. These endeavours will be impacted by global megatrends. The food wedges framework provides a simple but useful construct to begin to understand the likely contribution that different innovations might provide. It will be useful to further refine the food wedges framework. For example, the Food Security Committee of the International Union of Food Science and Technology (IUFoST) has been considering a version of the wedges framework that might be more reflective of the food value chain. It might also be useful to refine the framework in terms of the demand for a balance of nutrients for human health.

Nevertheless, it is hoped that this perspective and approach might facilitate dialogue between disciplines as well as providing a means to express the relative contribution or impact of a given research effort in food security to policy makers and other stakeholders.
